# Effects of Maternal Smoking on the Placental Expression of Genes Related to Angiogenesis and Apoptosis during the First Trimester

**DOI:** 10.1371/journal.pone.0106140

**Published:** 2014-08-28

**Authors:** Akihiro Kawashima, Keiko Koide, Walter Ventura, Kyoko Hori, Shin Takenaka, Daisuke Maruyama, Ryu Matsuoka, Kiyotake Ichizuka, Akihiko Sekizawa

**Affiliations:** Department of Obstetrics and Gynecology, Showa University School of Medicine, Shinagawa-ku, Tokyo, Japan; Medical Faculty, Otto-von-Guericke University Magdeburg, Medical Faculty, Germany

## Abstract

**Objective:**

Maternal cigarette smoking is reportedly associated with miscarriage, fetal growth restriction and placental abruption, and is paradoxically associated with a decreased risk of developing preeclampsia. In the present study, we investigated the gene expression levels of villous tissues in early gestation. We compared the expression levels of the genes related to angiogenesis and apoptosis in the villous tissues obtained from smoking and non-smoking pregnant women.

**Materials and Methods:**

We collected villous tissue samples from 57 women requesting surgical termination due to non-medical reasons at 6–8 weeks of gestation. The maternal cigarette smoking status was evaluated by the level of serum cotinine and patients were divided into active smokers and non-smokers by the serum cotinine level. The placental levels of VEGFA, PGF, FLT1, HIF1A, TP53, BAX and BCL2 mRNA were quantified by real time PCR.

**Results:**

The gene expression level of PGF and HIF1A in the active smoker group was significantly higher than that in the non-smoker group. We did not observe any significant differences in the VEGFA or FLT1 expression between the groups. In active smoker group, the gene expression levels of TP53 and BAX were significantly higher than those in the non-smoker group. The ratio of BAX/BCL2 mRNA in the active smoker group was significantly higher than that in the non-smoker group.

**Conclusions:**

Our findings revealed that smoking might affect the placenta during early pregnancy. Maternal cigarette smoking in early pregnancy may be associated with villus hypoxia, which may influence angiogenesis and apoptosis.

## Introduction

Cigarette smoking during pregnancy is a major public health concern in most industrialized nations. Despite a number of studies showing a decrease in the overall prevalence of smoking in women in the past 20 years, some studies have reported that 12–15% of all women smoke while pregnant [1]–[3]. It is acknowledged that exposure to tobacco during pregnancy modifies important aspects of the placental function. Previous studies have shown that there are placental complications linked to cigarette smoke exposure during pregnancy [4]. Smoking during pregnancy is associated with obstetric complications, spontaneous pregnancy loss, preterm premature rupture of the membrane, placental abruption and small-for-gestational-age birth [5]–[7]. On the other hand, cigarette smoking is associated with a decreased risk of preeclampsia [8], [9].

Marcoux et al [10] analyzed the data from a case-control study to evaluate effects of smoking before and during pregnancy. Smoking before pregnancy was not significantly related to preeclampsia. In a previous report, there was also no evidence that smoking before pregnancy affected the risk of developing hypertensive disease [11]. In the studies of the association between a reduced risk of preeclampsia and cessation of smoking during pregnancy, the protective effect of smoking for preeclampsia appears to continue even after the cessation of smoking [12], [13]. In addition to this protective effect, cigarette smoking increased the risk of small-for-gestational-age infants even after quitting smoking during the first trimester [14].

Several studies have suggested that cigarette smoking causes placental morphological changes. In term placentas, smoking in the third trimester broadened the basement membrane of the placenta, increased the collagen content of the villi and decreased vascularization [15]. Smoking also increased the syncytial knots and cytotrophoblast cells and decreased the vasculo-synctial membrane during the first trimester [16]. Jauniaux et al. showed that the areas presenting with syncytiotrophoblast necrosis were increased in smokers [17]. Cigarette smoking during pregnancy mediated pathological placental hypoxia and decreased cytotrophoblast proliferation [18]. These anatomical changes are all suggested to be associated with changes in placental functions.

Some studies suggested that maternal smoking affect the normal molecular biological reactions. Votavova et al. compared the transcriptome of the term placenta in smokers and non-smokers using a microarray analysis. Their findings demonstrated increased expression of genes related to coagulation and vasculogenesis and decreased expression of cell adhesion-related genes [19], [20]. In a recent study of the placental DNA methylation in term placentas, maternal smoking was found to deregulate the placental methylation in a CpG site-specific manner that correlated with meaningful alterations in gene expression among signature pathways [21]. Genbacev et al. showed that there was increased gene expression of VEGFA during the first trimester using immunostaining [22]. There have been a few reports that have investigated the molecular biological influence of maternal smoking on the villi at early gestation.

We hypothesized that maternal smoking affects angiogenesis and apoptosis in the villi during the first trimester, and may strongly influence subsequent spiral artery remodeling. We aimed to determine the association between maternal cigarette smoking and the expression of genes related to angiogenesis and apoptosis in the villi at early stages of pregnancy. Relying on the self-reported smoking status may lead to misclassification of the smoking status when carrying out studies on pregnancy [23], [24]. Therefore, in the present study, the maternal smoking status was defined by both a self-reported questionnaire and by the maternal serum cotinine level, which is the major metabolite of nicotine, which is widely used as a biomarker for tobacco exposure [25].

## Materials and Methods

### Study population

We recruited 57 women requesting elective termination of a pregnancy at 6–8 weeks of gestation. All participants were interviewed by a obstetrician to determine their smoking status before the termination. Additionally, an ultrasound examination was performed to confirm the fetal heart beat and the gestational age of the fetus (in weeks). The exclusion criteria included cases with multiple gestation, illicit drug use and preexisting medical conditions, such as diabetes, chronic hypertension and renal disease. The study was carried out in the Department of Obstetrics and Gynecology at Showa University School of Medicine (Tokyo, Japan) and approved by the Ethics Committee of human genomic analysis in Showa University School Medicine (144/2011). Written informed consent was obtained from each patient in this study.

### Blood sampling and cotinine analysis

Prior to the termination, blood samples were collected from the patients, and were centrifuged at 1,600 g for 10 min at 4°C. The resulting serum was transferred into plain polypropylene microtiter plates. The serum cotinine levels were quantified by using an enzyme-linked immune absorbent assay (ELISA; Cosmic Corporation, Tokyo, Japan) that had a detection limit of 0.6 ng/mL and an inter-assay variation of <7%. The participants were categorized as non-smokers if their serum levels were <1.0 ng/mL. The participants were categorized as active smokers if their levels were >5.3 ng/mL, since a serum cotinine cutoff of 3.0–5.3 ng/mL was previously recommended to separate smokers from non-smokers [26], [27]. The patients whose serum cotinine levels ranged between 1.0–5.3 ng/mL were excluded from this study.

### Placental sample collection

Placental tissue samples were obtained immediately after the surgical procedure and were washed with PBS to remove traces of maternal blood. The villous tissue was separated from the decidua using light microscopy. Each tissue sample was transferred to a tube containing 1.0 mL of RNAlater solution (RNA stabilization reagent, Qiagen, Hiden, Germany), was stored overnight at 4°C. After the reagent was removed, the samples were stored at −80°C until RNA isolation.

### RNA extraction

Frozen villous samples were thawed at 4°C. Then, 12 mg of the RNAlater preserved tissues were weighed on an analytical balance. Total RNA was extracted from the villous tissues using the RNeasy Mini Kit (Qiagen, Valencia, U.S.A) according to the manufacturer’s instructions. The purity of the RNA was evaluated with a NanoDrop ND-1000 spectrophotometer (Thermo Scientific Inc. Wilmington, U.S.A) by measuring the absorbance at 260 and 280 nm. OD260/280 ratios greater than 1.90 were considered to indicate that the samples were acceptable for further processing. All RNA samples met this purity requirement.

### Reverse transcription and quantitative real-time PCR

The extracted total RNA (2 µg) was immediately reverse-transcribed into cDNA using PrimeScript RT Master Mix (Takara Bio Inc., Shiga) according to the manufacturer’s instructions. The process was performed in the Veriti Thermal Cycler (Applied Biosystems Foster City, CA) with the following thermal conditions: 15 min at 37°C, followed by five min at 85°C. The real time quantitative PCR analysis was performed with the StepOnePlus Real-Time PCR System (Applied Biosystems, Foster City, U.S.A). Assay-on-Demand TaqMan primers and primers from Applied Biosystems were used. VEGFA (TaqMan Gene Expression Assay ID Hs00969450_m1), PGF (Hs00182176_m1), FLT1 (Hs01052936_m1), HIF1A (Hs00153132_m1), TP53 (Hs01034249_m1), BAX (Hs00180269_m1) and BCL2 (Hs00608023_m1). GAPDH (4310884E) was used as a reference gene. These selected genes were expressed under the following conditions: VEGFA and PGF are the main pro-angiogenic growth factors involved in placental vascular development. In addition, FLT1 is a receptor tyrosine kinase that binds to VEGFA and placental growth factor. HIF1A encodes hypoxia inducible factors that play an essential role in the cellular homeostatic response to hypoxia. TP53 encodes a tumor suppressor protein that induces apoptosis mediated by the expression of BAX (pro-apoptotic regulator) and repression of BCL2 (anti-apoptotic regulator).

The thermal cycling conditions were as follows: 95°C for 30 sec, followed by 40 cycles of 95°C for five sec and 60°C for 30 sec. All samples were analyzed in duplicate, and multiple negative water blanks were included in every analysis. The transcript numbers were determined from the linear regression of these standard curves. The gene expression levels were normalized to the level of GAPDH, and the relative expression of each gene is reported as a ratio (target gene/GAPDH).

### Statistical analysis

The data are presented as medians and interquartile ranges. The statistical significance of differences was assessed by the Wilcoxon rank-sum test. Categorical variables were compared by Fisher’s exact test. All analyses were carried out using the JMP version 10.0.2 software program (SAS Institute, Cary, NC). A value of *p*<0.05 was considered to be statistically significant.

## Results

The cotinine levels in the serum sample were assayed in 57 cases. The participants were divided into two groups: a non-smoker group (n = 32), with a serum cotinine level <1.0 ng/mL and an active smoker group (n = 20), with a serum cotinine level >5.3 ng/mL. The patients with a serum cotinine level between 1.0–5.3 ng/mL were excluded (n = 5). The clinical background available for each study group, the gestational age in weeks, the self-reported smoking status and the cotinine concentrations are presented in Table1. There were no significant differences in maternal age, gestational age, systolic blood pressure, diastolic blood pressure and crown-rump length between the groups. There were significant differences in the self-reported smoking status and the serum cotinine level between the two groups, as would be expected.

**Table 1:** The background and cotinine levels in the study group.

	Non-smoker (n = 32)	Active smoker (n = 20)	p value
Age (years old)	26 (24–34)	30 (26–35)	0.206^a^
Self-reported smoker (%)	0%	85.0%	<0.001^b^
Cigarettes (/Day)	0 (0–0)	10 (8–20)	<0.001^a^
Gestational age (days)	52 (47–58)	51 (48–57)	0.954^a^
Systolic blood pressure (mmHg)	102 (96–113)	112 (96–121)	0.152^a^
Diastolic blood pressure (mmHg)	56 (49–65)	66 (50–75)	0.212^a^
Crown-rump length (mm)	11 (5.9–17.5)	11.9 (6.0–15.2)	0.940^a^
Serum cotinine (ng/mL)	0.42 (0–0.60)	180 (116–330)	<0.001^a^

There were no significant differences in the maternal age, gestational age, systolic blood pressure, diastolic blood pressure and crown-rump length between the non-smokers and the active smokers. The data are presented as medians and quartiles.

a *p* values are based on Wilcoxon rank-sum test.

b *p* values were obtained by Fisher’s exact test.

**Figure 1:**
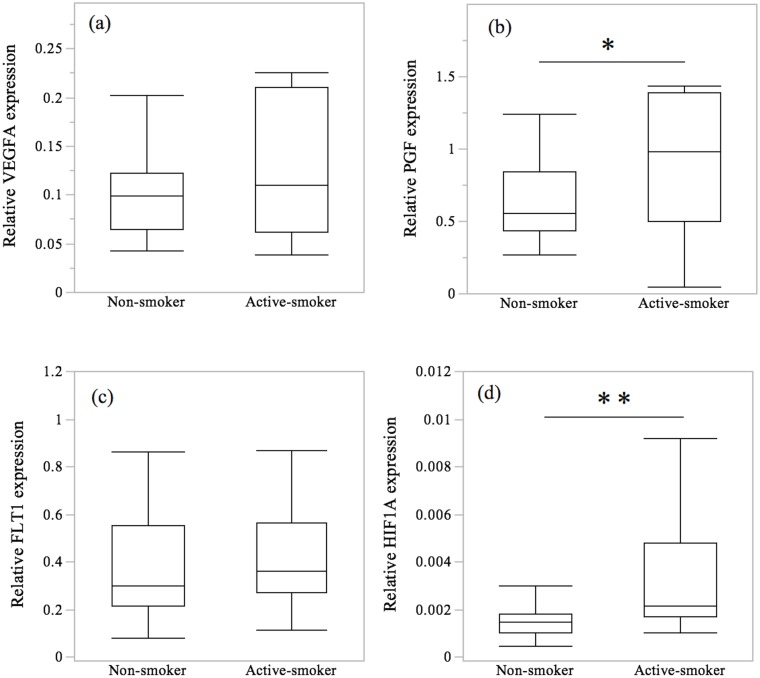
The expression of angiogenesis-related genes. Box plots of the mRNA transcripts of VEGFA, PGF, FLT1 and HIF1A. (**a–d**) By using real-time quantitative PCR, we found significantly increased expression of PGF and HIF1A mRNA in the villi in the active-smoker group compared to the non-smoker group. The values are relative to the expression of GAPDH. The central bars represent the median values, boxes represent the interquartile ranges and whiskers represent the 90th and 10th percentiles. *, *p*<0.05; **p<0.01, in the comparison between non-smokers and active-smokers.

Figure 1 shows the expression of the selected genes related to angiogenesis. There were no significant differences in the expression levels of VEGFA or FLT1 mRNA between the non-smoking and active smoking group. In contrast, the relative abundance of PGF mRNA and HIF1A was greater in active smokers than in non-smoker (*p* = 0.025 and *p* = 0.003).

**Figure 2:**
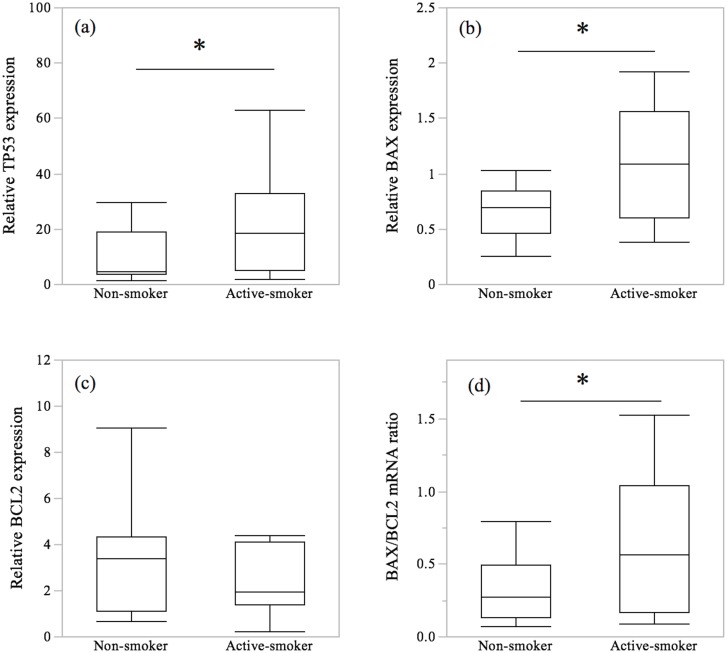
The expression of apoptosis-related genes. Box plots of the mRNA transcripts of TP53, BAX and BCL2, and the ratio of BAX/BCL2 mRNA. (**a–c**) By using real-time quantitative PCR, we found significantly increased expression of TP53 and BAX mRNA in the villi in the active smoker group compared to the non-smoker group. (**d**) Maternal smoking exposure increased the ratio of BAX/BCL2 mRNA. The values are relative to GAPDH. The central bars represent the median values, boxes represent the interquartile ranges and whiskers represent the 90th and 10th percentiles. *, *p*<0.05, in a comparison between non-smokers and active smokers.

The gene expression levels of TP53 and BAX were significantly higher in the active smoking group compared with the non-smoking group (*p* = 0.046 and *p* = 0.042, respectively). We found no significant differences between active smokers and non-smokers in the mRNA expression levels of BCL2. Overall, the mRNA ratio of BAX/BCL2 in the active smoker group was significantly increased compared to that in the non-smoker group (*p* = 0.022) (Figure 2).

## Discussion

Maternal smoking during the first trimester is associated with a decreased risk of preeclampsia and an increased risk of having an infant small-for-gestational-age, therefore smoking might alter the placental expression of genes related to the pathogenesis of preeclampsia and small-for-gestational-age. In this study, we found that the mRNA expression level of PGF, a pro-angiogenic gene, increased in the villous tissue of smokers at six to eight weeks of gestation. Moreover, we also discovered that the mRNA expression levels of pro-apoptotic genes, TP53 and BAX, were increased, as was the mRNA ratio of BAX/BCL2, suggesting that the apoptotic potential of the cells was also increased. These findings suggest that maternal smoking exposure affected the pathophysiology of the villi during the first trimester by altering the angiogenesis and apoptosis.

The effects of maternal smoking on the placental transcriptome expression probably start in the first trimester. We previously reported that we could not observe any significant effects of maternal smoking in the expression levels of genes related to angiogenesis in the villous trophoblasts obtained from tissue samples obtained at six to seven weeks of gestation [28]. Since the number of samples was small in that study, in the present study, we increased the number of samples and reexamined the effects of maternal smoking on the placenta. Our first trimester villous samples provided a unique opportunity to obtain information regarding the expression of genes related to angiogenesis and apoptosis in cases with or without maternal smoking. The current knowledge regarding the effects of maternal smoking on the placenta suggests that exposure of placental villous explants to cigarette smoke extract results in a pro-angiogenic state, with a relative abundance of PGF, and this reverses the change that are seen in preeclampsia, and may explain the reduction of preeclampsia in smokers [29]. Numerous studies have shown that the serum protein level of PGF levels were higher in smokers compared to non-smokers during all three trimesters [30]–[32]. PGF and FLT1 play roles in the mobilization of mesenchymal endothelial precursor cells that contribute to vasculogenesis [33], and they are highly expressed by invasive cytotrophoblasts [34]. Gene expression studies from chorionic villous sampling in women who subsequently developed preeclampsia demonstrated an increasing expression of VEGFA and a decreasing expression of PGF [35], [36]. This suggested that the unbalanced angiogenesis and altered decidua/placental vascular adaptation during the first trimester might be the trigger for the pathogenesis of preeclampsia. At this point, our findings in active smokers suggest that certain elements or products of cigarette smoke may alter the placental gene profile and contribute to the reduced incidence of preeclampsia.

To determine whether the mitochondrial pathway is affected in responses to maternal cigarette smoking, we analyzed the gene expressions of changes in these pro- and anti-apoptotic genes, and in HIF1A gene in the villi from patients with or without a smoking habit. Our studies demonstrated that maternal smoking increased the expression of HIF1A, BAX and TP53 mRNA, and also increased the ratio of BAX/BCL2 mRNA in the villi.

Apoptosis occurs via death receptor-dependent or mitochondrial pathways. The death receptor-dependent pathway is triggered by the activations of death receptors, such as Fas and TRAIL, which activate the initiator caspase 8, followed by the cleavage of the executioner caspase 3. The mitochondrial pathway is regulated by members of the Bcl-2 family of proteins under the control of p53. It is known that p53 induces the expression of pro-apoptotic genes, such as BAX, and that p53 directly binds Bcl-2 at the mitochondrial membrane. Disruption of the mitochondrial membrane potential results in the release of pro-apoptotic factors, such as cytochrome c, from the mitochondria into the cytosol, which activate caspase 9 and then caspase 3. Caspase 3 then catalyzes the degradation of proteins involved in vital cellular processes [37], [38].

It has been shown that components of cigarette smoke are pathogenic during pregnancy, resulting in spontaneous abortion, intrauterine growth restriction and low birth weight. Cigarette smoking was reported to be associated with the cell death pathway. Over 4,000 different chemicals are present in cigarette smoke. Drukteinis et al. observed that benzo[a]-pyrene, which is a major toxicant in cigarette smoke, activated a p53-dependent cell death pathway, providing evidence of oxidative stress in a trophoblast cell line [39]. Higher expression of BAX was observed in mouse ovaries after exposure to benzo[a]-pyrene [40]. Moreover, using ovarian cell cultures from mouse fetuses, fetal oocyte apoptosis and increasing expression of BAX were observed after the addition of benzo[a]-pyrene [41]. In normal term fetal membrane explant cultures, the effects of cigarette smoke extract was found to be potentially mediated by apoptosis occurring via a p53-dependent pathway [42], [43].

A series of *in vitro* studies have revealed the pro-apoptotic effect of hypoxia in human cytotrophoblasts; this effect involves the increased internucleosomal cleavage of DNA, the up-regulation of pro-apoptotic proteins, such as p53 and Bax, and the decreased expression of the anti-apoptotic Bcl-2 proteins [44], [45]. Increased apoptosis in the trophoblasts may contribute to impaired placental function and suboptimal fetal growth, and p53 may play a pivotal and complex role in regulating the trophoblast cell turnover in response to hypoxic stress [46]. It has been considered that p53 is the most important downstream effector of HIF1A, which induces apoptosis *in vitro* [47]. A study by Blouin et al. in macrophages demonstrated that the exposure to inflammatory stimuli under normoxic conditions can drive the transcription of a similar set of stress response genes as are stimulated by HIF1A under hypoxic conditions [48]. Under hypoxic conditions, the increase in HIF1A may lead to a direct increase in p53, which may be one of the factors involved in the pathogenesis of hypoxia [49]. Our findings support the idea that maternal smoking increases the apoptotic changes via the mitochondrial pathway in the villi during the first trimester.

**Figure 3:**
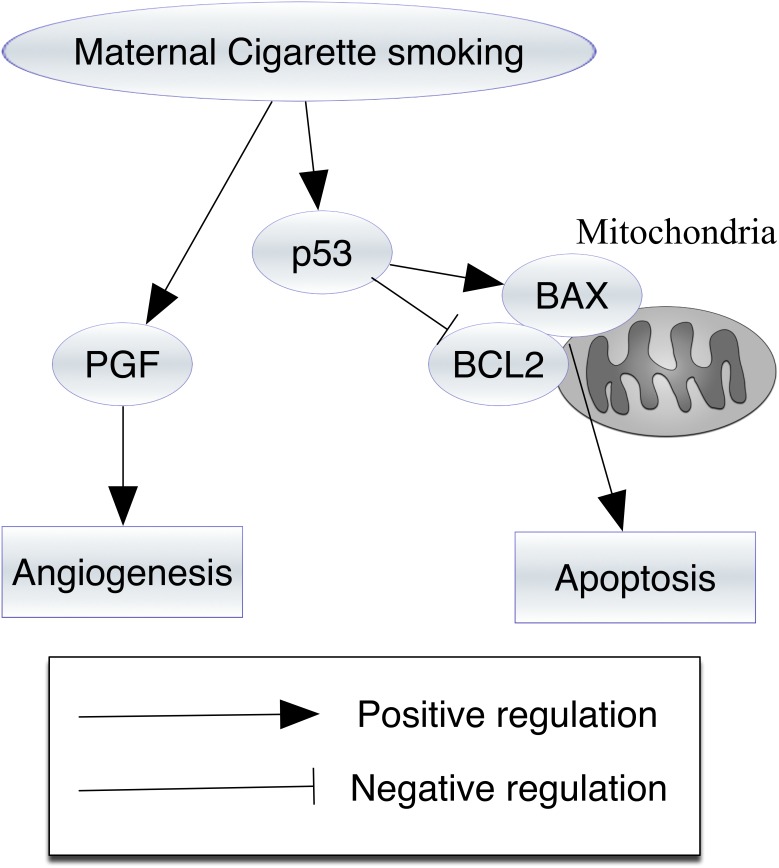
A schematic diagram showing that increased angiogenesis and apoptosis in the villi during the first trimester is associated with maternal cigarette smoking. Maternal cigarette smoking seems to increase the expression of placental growth factor (PGF), which promotes angiogenesis. In addition, maternal cigarette smoking seems to activate the p53 pathway. The p53 activation can induce the expression of a pro-apoptotic Bcl-2 family (BCL2-associated X protein, BAX) and inhibit that of an anti-apoptotic member (B-cell lymphoma 2, BCL2). The increased ratio of BAX to BCL2 may contribute to apoptosis.

The effects of maternal smoking on the morphology of the villi are well recognized. We found that maternal cigarette smoking might induce angiogenesis via PGF and apoptosis via the mitochondrial pathway (Figure 3). In this study, the specific abnormalities that are caused by maternal cigarette smoking provide additional insights into the genes and processes that are most crucial for the formation of the fetomaternal interface in human pregnancy. Our findings offer speculative information regarding the effects of smoking during pregnancy.

There are several potential limitations associated with the present study, such as the small sample size and the limited gene expression studies. Further examinations about the influence of maternal smoking on the epigenome and proteome will be needed.
